# KNOWLEDGE OF AGEISM AND ATTITUDES ABOUT AGING AS A CORE COMPETENCY FOR HEALTH PROFESSIONALS

**DOI:** 10.1093/geroni/igz038.3067

**Published:** 2019-11-08

**Authors:** Sarah Marrs, Tracey Gendron, Leland Waters, Jenny Inker, Maddie McIntyre

**Affiliations:** Virginia Commonwealth University, Richmond, Virginia, United States

## Abstract

Senior mentoring programs have been established that provide medical students exposure to a community-dwelling older adult mentor with whom they meet multiple times throughout the program. The goal of these programs is to expose students to healthy older adults, increase knowledge of geriatrics, and prepare them to care for an aging population. However, even while participating in a senior mentoring program, health professions students still demonstrate some discriminatory language towards older adults (e.g., Gendron, Inker, & Welleford, 2018). In fact, research suggests ageist practices occur, intentionally or not, among health professions in disciplines such as medicine, nursing, and social work and even within assisted and long-term care facilities (e.g., Bowling, 1999; Dobbs et al., 2008; Kane & Kane, 2005). We evaluated a senior mentoring program to gauge the impact of a new pedagogical approach and to gain a deeper understanding of the learning gained in relation to ageism and elderhood. This qualitative content analysis explored first-year medical students’ opinions of their own aging and attitudes towards caring for older adults. Students (n = 216) participating in a brief curriculum model of a senior mentoring program responded to the following open-ended prompts before and after the program: 1) How do you feel about your own aging?; 2) How do you feel about working with older adult patients after you complete your medical training? Responses suggest that students’ views of their own aging and views towards towards working with older patients are positively impacted by their experiences in the senior mentoring program.

